# RETRACTED: Cordaro et al. Role of Etanercept and Infliximab on Nociceptive Changes Induced by the Experimental Model of Fibromyalgia. *Int. J. Mol. Sci.* 2022, *23*, 6139

**DOI:** 10.3390/ijms27031519

**Published:** 2026-02-04

**Authors:** Marika Cordaro, Rosalba Siracusa, Ramona D’Amico, Tiziana Genovese, Gianluca Franco, Ylenia Marino, Davide Di Paola, Salvatore Cuzzocrea, Daniela Impellizzeri, Rosanna Di Paola, Roberta Fusco

**Affiliations:** 1 Department of Biomedical, Dental and Morphological and Functional Imaging University of Messina, Via Consolare Valeria, 98125 Messina, Italy; 2 Department of Chemical, Biological, Pharmaceutical and Environmental Sciences, University of Messina, Viale Ferdinando Stagno D’Alcontres 31, 98166 Messina, Italytgenovese@unime.it (T.G.);; 3 Department of Veterinary Sciences, University of Messina, 98168 Messina, Italy; 4 Department of Clinical and Experimental Medicine, University of Messina, Via Consolare Valeria, 98125 Messina, Italy

The journal retracts the article titled “Role of Etanercept and Infliximab on Nociceptive Changes Induced by the Experimental Model of Fibromyalgia” [1], cited above.

Following publication, concerns were brought to the attention of the Editorial Office regarding the integrity of a number of images presented in this publication [1].

Adhering to our standard procedure, the Editorial Office and Editorial Board conducted an investigation that confirmed the duplication of a number of panels contained within Figure 5, presenting different experimental conditions. Following discussions with the authors, the Editorial Board was unable to accept the authors’ explanation or verify the validity of the above-mentioned images through evaluation of the supporting raw materials provided. Consequently, the Editorial Board has lost confidence in the reliability of the findings and has decided to retract this publication as per MDPI’s retraction policy (https://www.mdpi.com/ethics#_bookmark30).

This retraction was approved by the Editor-in-Chief of the journal *IJMS*.

The authors do not agree with this retraction.
